# Pharmacokinetic and pharmacodynamic interactions: from theory to practice

**DOI:** 10.1192/j.eurpsy.2026.10304

**Published:** 2026-07-31

**Authors:** M. Stuhec

**Affiliations:** Clinical Pharmacy and & Pharmacology, Medical Faculty Maribor, University Maribor, Maribor, Slovenia

## Abstract

**Abstract:**

Drug–drug interactions (DDIs) are significant pharmacokinetic and pharmacodynamic consequences of concomitant treatment with two or more agents. DDIs are categorized as potential or clinically relevant. While many DDIs are identified as potential, only approximately 5% become clinically relevant. Various strategies—including the use of DDI checking tools, collaboration with clinical pharmacists, and systematic medication reviews—can reduce the occurrence of potential DDIs and, consequently, the risk of clinically significant DDIs. The speaker will present basic methods for detecting potential DDIs using a clinical case and will discuss the most recommended approaches to reducing clinically relevant DDIs in everyday clinical practice.

**Disclosure of Interest:**

None Declared

